# Effects of the complete replacement of fish oil with linseed oil on growth, fatty acid composition, and protein expression in the Chinese mitten crab (*Eriocheir sinensis*)

**DOI:** 10.1186/s12953-018-0135-7

**Published:** 2018-03-15

**Authors:** Banghong Wei, Zhigang Yang, Yongxu Cheng, Jianyi Wang, Junyu Zhou

**Affiliations:** 1 Key Laboratory of Exploration and Utilization of Aquatic Genetic Resources, Ministry of Education, Shanghai Ocean University, Shanghai, 201306 China; 2 Centre for Research on Environmental Ecology and Fish Nutrition (CREEFN) of the Ministry of Agriculture, Shanghai Ocean University, Shanghai, 201306 China; 3 National Demonstration Center for Experimental Fisheries Science Education (Shanghai Ocean University), Shanghai, 201306 China

**Keywords:** *Eriocheir sinensis*, Proteome, Dietary lipid, Growth performance, Digestive enzyme activity, Fatty acids composition

## Abstract

**Background:**

The finite marine resources make it difficult for us to obtain enough fish oil (FO) used in aquatic feeds. Another sustainable ingredients should be found to substitute FO. The effects of replacing FO with vegetable oil have been studied in a variety of crustaceans, but most studies have focused on the phenotypic effects. Little is known about the mechanisms of the effects.

**Methods:**

To understand the molecular responses during the replacement of FO in *Eriocheir sinensis*, we investigated the effects of feeding FO or linseed oil (LO) on growth performance, digestive enzyme activity, fatty acid composition and protein expression in *E. sinensis*. Twenty-four juvenile crabs were fed diets containing FO or LO for 112 days. Weight, carapace length and width were recorded. Fatty acid composition of the diets and the hepatopancreas and protein expression in the hepatopancreas were analyzed.

**Results:**

Growth performance and molting interval were unchanged by diet. Crabs fed FO and LO had same activity of lipase and amylase, but comparing with crabs fed LO, crabs fed FO had higher trypsin activity and lower pepsin activity. Hepatopancreas fatty acid composition changed to reflect the fatty acid composition of the diets. In total, 194 proteins were differentially expressed in the hepatopancreas between the diets. Expression of heat shock proteins was higher in crabs fed LO. Expression of fatty acid synthase, long-chain fatty acid transport protein 4, acyl-CoA delta-9 desaturase, and fatty acid-binding protein 1, was higher in crabs fed FO.

**Conclusions:**

The substitution of FO with LO didn’t have any effects on the growth and molting of mitten crab, but could significantly decrease the ability of mitten crab to cope with stress. The high content of HUFAs in the hepatopancreas of mitten crab fed FO is due to the high abundance of the proteins relative to the transport of the HUFAs. These findings provide a reason of the high content of EPA and DHA in crabs fed with FO, and provide new information for the replacement of FO in diets of mitten crab.

**Electronic supplementary material:**

The online version of this article (10.1186/s12953-018-0135-7) contains supplementary material, which is available to authorized users.

## Background

The Chinese mitten crab, *Eriocheir sinensis*, is an economically important crab species in China [1] with 812,103 tons of mitten crab produced by the Chinese aquaculture industry in 2016, the highest production weight for fresh water crustaceans (China Fisheries Yearbook 2017). Mitten crab contains a high content of highly unsaturated fatty acids (HUFAs) such as eicosapentaenoic acid (EPA, 20:5n-3) and docosahexaenoic acid (DHA, 22:6n-3) [2]. The commercial and nutritional importance of mitten crab has stimulated research into the nutrition of the crab.

Dietary lipids are the main source of energy and provide essential fatty acids, phospholipids, and fat-soluble vitamins for crustaceans [3–5]. At present, as essential fatty acids, the HUFAs in diets of mitten crab are provided by fish oil (FO). However, the sharp decline of wild fisheries has driven the search for alternative sources of fat to substitute FO in the crustacean diet [6, 7]. But different effects were obtained in different studies [6, 8–13]. However, it is now well established from a variety of studies that the fatty acid composition of the hepatopancreas and muscle in mitten crab is correlated with the fatty acid composition of the diet. FO is rich in HUFAs, while vegetable oils mostly contain α-linolenic acid (ALA, 18:3n-3) and linoleic acid (LA, 18:2n-6), which are the biological precursors of HUFAs, such as EPA and DHA [14]. As far as we know, most freshwater fish have the ability to synthesize HUFAs using ALA and LA, whereas marine fish lack the biosynthesis capacity [15–17]. At present, limited knowledge is known about the HUFA biosynthesis capacity in mitten crab, the mechanisms explaining the effects of the replacement of FO on the fatty acid composition require further investigation.

High-throughput mass spectrometric proteomic technologies can now analyze the whole proteome expressed in a particular cell or organ [18]. Compared to large-scale transcriptome analysis that analyzes changes in gene transcription, the proteome reveals changes in the amounts of individual protein representing functional changes in the cell or organ [19, 20]. Thus proteomic analysis become an useful tool which can be used to characterize and understand the mechanisms of the responses to dietary changes [21].

Parallel reaction monitoring (PRM) is a new developed method in targeted mass spectrometry equipped with a quadrupole-equipped orbitrap [22]. And it has been widely used in the quantification and detection of the target proteins [23, 24].

In this study, to understand the mechanisms explaining the effects of the replacement of FO on the fatty acid composition, we analyzed the effects of two diets containing either fish oil or linseed oil on the growth performance, fatty acid composition, and protein expression of the hepatopancreas in mitten crab. And the alterations in protein expression levels were verified using quantitative real-time reverse-transcription (RT-PCR) and PRM.

## Methods

### Experimental diets

Two isonitrogenous and isolipidic purified experimental diets were formulated using FO (fish oil) or LO (linseed oil) as a source of lipids. The raw material was selected by 80 meshes after grinding. And then were blended and moistened with water. The mixture was further pelletized using a pelletizer. The diets were formed into 1.5 mm (diameter) pellets, and stored at − 20 °C until used. The composition and formulation of the experimental diets are detailed in Table 1.

**Table 1:** Composition and calculated proximate composition of the diet

Ingredients (%)	Diets group	
	FO	LO
Casein	41	41
Cellulose	4	4
Wheat flour	28.65	28.65
Carboxymethylcellulose (CMC)	4	4
Yeast extract	5	5
Lysine	0.15	0.15
Glycine	0.5	0.5
Vitamin C (99.7%)	0.5	0.5
Vitamin E (97%)	0.1	0.1
Phospholipid (99%)	3	3
Cholesterol	0.5	0.5
Inositol	0.6	0.6
Choline chloride (50%)	1	1
Mineral premix^a^	3	3
Vitamin premix^b^	2	2
Fish oil	6	0
Linseed oil	0	6
Proximate composition (percentage of dry weight)		
Crude protein	39.26	39.44
Crude lipid	9.75	9.47
Ash	5.68	5.72

^a^Mineral premix: 1 kg diet contained Ca(H_2_PO_4_)_2_, 10 g; MgSO_4_·7H_2_O, 2.4 g; KCl, 4.5 g; NaCl, 2.1 g; FeSO_4_·H_2_O, 155 mg; CuSO_4_·5H_2_O, 40 mg; ZnSO_4_·H_2_O, 80 mg; MnSO_4_·H_2_O, 30 mg; KI, 11.7 mg; CoCl_2_·6H_2_O, 4.8 mg; Na_2_SeO_3_, 2.4 mg

^b^Vitamin premix: 1 kg diet contained vitamin A, 10000 IU; vitamin D, 2500 IU; vitamin K, 64 mg; thiamin, 60 mg; riboflavin, 250 mg; pyridoxine, 60 mg; calcium pantothenate, 240 mg; niacin, 60 mg; folic acid, 12 mg; biotin, 50 mg; cyanocobalamin, 4 mg

### Experimental animals and feeding trials

Juvenile Chinese mitten crabs were obtained from the Chongming research base of Shanghai Ocean University. After a week acclimation, 24 healthy male crabs were randomly assigned into 2 groups with 12 crabs in each group. The initial weight of crabs in FO and SO were 2.13 g, initial carapace length and initial carapace width were shown in Table 2. Each crab was cultivated in a single plastic box (36 cm × 18 cm × 18 cm). The two groups were randomly assigned to one of the two experimental diets and were fed once a day at 13:00 for 112 days; the experimental diets were offered as 5% of body weight, and were adjusted by the growth. Uneaten feed was removed using a siphon tube after 2 h and oven-dried for analysis of the feed coefficient. During the experiment, water was exchanged once daily with 1/3–1/2 of the tank volume, and was aerated throughout the feeding trial to maintain dissolved oxygen > 5 mg/L. The photoperiod was approximately 12 L:12D. Natural temperature of the water during the feeding trials was 24.5–30.0 °C. Water quality parameters were monitored 2–3 times a week to maintain at pH 8.0 ± 0.4 and total ammonia nitrogen < 0.01 mg/L.

**Table 2:** Effects of feeding crabs FO or LO diets on growth performance of juvenile mitten crab (means ± SD)

	Groups	
	FO	LO
Survival rate (%)	75	75
Initial weight (g, *n* = 12)	2.13 ± 0.08	2.13 ± 0.06
Initial carapace length (mm, *n* = 12)	15.28 ± 0.14	15.21 ± 0.14
Initial carapace width (mm, *n* = 12)	17.24 ± 0.20	17.22 ± 0.27
Final weight (g, *n* = 9)	5.39 ± 0.48	5.60 ± 0.80
Final carapace length (mm, *n* = 9)	20.42 ± 0.87	20.59 ± 1.14
Final carapace width (mm, *n* = 9)	22.17 ± 0.80	22.75 ± 1.19
Weight gain (*n* = 9, %)	153.30 ± 24.81	164.50 ± 45.54
Specific growth rate (*n* = 9, %)	0.82 ± 0.09	0.85 ± 0.15
Hepatosomatic index (*n* = 9, %)	7.75 ± 0.26	8.03 ± 0.34
Feed conversion ratio	2.16 ± 0.11	2.02 ± 0.08

### Measurement of growth performance and sample collection

Each crab was weighed and the length and width of the carapace was measured at the beginning and end of the study. And the intermolt duration of each crab were recorded for the analysis of molting. Three crabs which have weight around average weight were selected, and the hepatopancreas was collected from three crabs after being fasted for 24 h. The collected samples were immediately frozen in liquid nitrogen and then stored at − 80 °C for fatty acids composition and proteomic analysis.

The parameters were measured in the following methods:

Surviving rate (S, %) = final number of crabs/initial number of crabs × 100;

Weight gain (WG, %) = [(final body weight - initial body weight)/initial body weight] × 100;

Specific growth rate (SGR, %) = (Ln final body weight - Ln initial body weight) × 100/numbers of days;

Feed conversion ratio (FCR) = feed consumed after correction of dissolution/ (final body weight - initial body weight);

Hepatosomatic index (%) = weight of hepatopancreas/final body weight × 100.

### Measurement of digestive enzymes

Hepatopancreas of three crabs in each group were randomly selected for the analysis of digestive enzymes. Sample preparation were performed following the manufacturer’s instructions for the measurement of trypsin (Code: A080–2), pepsin(Code: A080–1), amylase (Code: C016–1) and lipase (Code: A054–1) (Nanjing Jiancheng Bioengineering Institute, http://www.njjcbio.com/). A trypsin and pepsin unit are defined as the 0.003 changes in absorption value caused by enzyme activity in per mg protein per min under the assay conditions. A amylase unit is defined as enzyme activity per mg protein that catalyzed the hydrolyzation of 10 mg starch in 30 min. A lipase unit is defined as the consumption of 1 mol matrix that 1 g tissue protein reacted with the matrix at 37 °C for 1 min.

### Fatty acid analysis

The fatty acids composition of experimental diets and hepatopancreas from three crabs was determined following the methods used by Folch [25]. The methyl-esterification of fatty acids was performed according to Morrison [26]. The fatty acid compositions were analyzed using an Agilent 5977 GC-MS chromatograph. Injection and detector temperature were set at 200 °C and 250 °C, respectively. The oven temperature was programmed to increase from an initial temperature of 70 °C at a rate of 50 °C/min, and held at 140 °C for 1 min, then increase from 140 °C to 180 °C at 4 °C/min, and held at 180 °C for 1 min. At last, increase from 180 °C to 225 °C at 3 °C/min, held at 225 °C for 30 min until no peak appeared. The percent area under each peak of each chromatogram was quantified to determine the quantity of each fatty acids.

### Protein extraction and protein quantification, quality analysis

Samples were prepared for proteome analysis according to the methods detailed in Wisniewski [27]. Hepatopancreas sample was ground in liquid nitrogen using a refrigerated mortar. After fully grinding, the ground powder was transferred to 0.4 ml protein lysate [8.0 M Urea, Sigma; 10 mM, DL-Dithiothreitol (DTT), Genview; 100 mM Tris-HCl; 1 × protease inhibitor (Roch); pH, 8.0], and was further ground until large block tissue disappeared. Then the sample was sonicated 5 times for 2 s each using an ultrasonic smash VCX800 (Sonics). After 20 min in ice, the ultrasonic broken sample was centrifuged at 10,000 g for 30 min at 4 °C and the supernatant was transferred into a new tube stored at − 80 °C. The concentration of extracted proteins was quantified with biotechnology grade BSA protein as a quantification standard [28]. Protein quality were determined by sodium dodecyl sulfate polyacrylamide gel electrophoresis (SDS-PAGE).

### Proteolysis and LC-MS/MS

Samples were digested using trypsin (Promega, USA) in a ratio of protein:trypsin at 100:1. The solution was incubated at 37 °C for more than 12 h. Sample digests were analyzed in triplicate. Samples were separated using Nano high performance liquid chromatography (HPLC) Ultimate 3000 (Thermo Fisher Scientific, USA). The mobile phases A and B were 0.1% formic acid in water, and 80% acetonitrile (ACN) with 0.1% formic acid in water, respectively. The column was equilibrated with 95% buffer A. The sample were injected from an autosampler onto a C18 trap column (Bangfei Bioscience; 3 m, 0.10 × 20 mm), and were eluted onto an analytical C18 column (Bangfei Bioscience; 1.9 m, 0.15 × 120 mm) at a flow rate of 600 nL/min. Proteins were identified by mass spectrometry analysis, performed using Q Exactive HF (Thermo Fisher Scientific, USA) coupled to the Nano HPLC. The instrument settings were as follows: the resolution was set to 120,000 for MS scans, and 15,000 for the MS/MS scans. The MS scan range was from 300 to 1400 m/z. The MS AGC target was set to 3 × 10^6^ counts, whereas the MS/MS AGC target was set to 5 × 10^4^. The isolation window was set to 1.6 m/z.

### Data analysis and protein quantification

The mass spectrometry data were analyzed by Proteome Discoverer, version 2.0 (Thermo Fisher Scientific) and in-house Mascot, version 2.2 (Matrix Science) against the database from the transcriptome of mitten crab obtained before the proteomic analysis with peptide false discovery rate (FDR) ≤ 0.01. The identification of proteins by Proteome Discoverer was performed using the following parameters: trypsin was the digesting enzyme, and 2 max missed cleavages were allowed. Fixed modification was carbamidomethylation of cysteines, and the oxidation of methionines and acetyl of protein N-term were allowed as a variable modification. Peptide mass tolerance and fragment mass tolerance were ±15 ppm and 20mmu, respectively. The quantification of the proteins was calculated according to the spectral area of the peptide and protein [29]. Proteins were regarded as differentially expressed between diet groups when the fold change (FC) of quantitative ratios was ≥ 2 (upregulated), or ≤ 0.5 (downregulated).

### Bioinformatics analysis

As a non-model organism, the bioinformatics analysis of the proteome in the present study was performed according to the annotation of the transcriptome [30] . ID conversion of the proteins was performed using the annotation, and then Gene ontology (GO) and EuKaryotic Orthologous Groups (KOG) annotation were assigned to the proteins identified in the present study.

### Validation of the differentially expressed proteins

RT-PCR was used to verify whether six proteins that were significantly increased in LO-fed crabs were also increased in expression at the mRNA level. FC of the six proteins are in Table 3. The primer sequences are listed in Table 4. Gene expression was normalized to 18S ribosomal RNA (18S). Total RNA of three crabs in each group was extracted from the hepatopancreas using TRIzol (Invitrogen) according the manufacturer’s instructions. Then, first-strand cDNA was synthesized using PrimeScript RT Master Mix (TaKaRa Bio, Japan). The RT-PCR was carried out in a 7500 Real-Time PCR System (Applied Biosystems, USA) using the template above following the manufacturer’s instructions for SYBR Premix Ex Taq (TaKaRa Bio, Japan). The RT-PCR was carried out in a total volume of 10 μL: 5 μL 2× SYBR Premix Ex Taq, 0.2 μL 50 × ROX Reference Dye II, 1 μL diluted cDNA mix, 0.2 μL each primer (10 μM), and 3.4 μL sterile distilled water. Three replicates were carried out for each sample. The transcript levels were calculated using the comparative threshold cycle (2^−ΔΔCt^) formula. More information about the formulation of the comparative threshold cycle (2^-ΔΔCt^) formula was referred to Schmittgen [31].

**Table 3:** Validation of differentially expressed proteins in proteomic analysis using PRM analysis

Protein	FC (LO/FO) in proteomic	*P*-value	FC (LO/FO) in PRM	*P*-value
HSP70	5.81	0.0075	5.32	0.0067
Tpi	2.30	0.0213	3.05	0.0134
AM	4.32	0.0019	6.78	0.0502
Cyc	2.30	0.0167	2.68	0.0090
HS6	2.09	0.0015	1.73	0.0057
Cat	2.61	0.1313	2.26	0.0059

**Table 4:** The primers used for verification of the differentially expressed genes

Gene name	Primer sequence (5′ → 3′)	Product length (bp)
HSP70-F	CTACACCTCCATCACCCGT	105
HSP70-R	TTGTCCATCTTGGCATCAC	
Tpi-F	AGGTTGTGGTGGGATGTC	178
Tpi-R	TCTGAGTGTCCAAGGATGA	
AM-F	TCAAGGACGACCTCAAGAA	132
AM-R	CCCTCAGCCGAAGACAC	
Cyc-F	GGAGGCACAGAAAGAAGC	129
Cyc-R	TAGACGAGAACCGAACGC	
HS6-F	AGCCCAGATGACCCAAACTC	214
HS6-R	CTGATGGTGGACCCAGAAGA	
Cat-F	CCAAAGGTCTTACTTCGCC	140
Cat-R	CCGTGGTTTTATTGCCAA	
18S-F	TCCAGTTCGCAGCTTCTTCTT	90
18S-R	AACATCTAAGGGCATCACAGA	

*HSP70* heat shock protein 70, *Tpi* triosephosphate isomerase, *AM* alpha 2-macroglobulin, *Cyc* cyclophilin A, *HS6*: hemocyanin subunit 6, *Cat* cathepsin C, *18S* 18S ribosomal RNA

And the selected proteins were further quantified by PRM-MS analysis at Beijing BangFei Bioscience Co., Ltd. (Beijing, China, http://www.bangfeibio.com/). The proteins were prepared following the proteomic analysis above. Then the peptide was introduced into the mass spectrometer. The raw data obtained were then analyzed using Proteome Discoverer 2.0 (Thermo Fisher Scientific). Skyline software was used for quantitative data processing.

### Statistical analysis

All the results were presented as means ± SD and analyzed by Student’s t-test. Statistical analysis was performed using SPSS Statistics V22.0 (IBM Corporation, NY, USA). Data were considered to be statistically significant when *P* < 0.05.

## Results

### Growth performance of juvenile mitten crab fed either FO or LO diets

The total survival rate was 75% in both the FO and LO diet groups (Table 2). Initial weight, carapace length, and carapace width was the same for crabs in both groups (Table 2). There were no differences in the weight, carapace length, carapace width, weight gain, special weight gain, and hepatosomatic index at the end of the experiment (Table 2).

### Intermolt of juvenile mitten crab fed either FO or LO diets

During the experiment, all the crabs had successfully molted for 3 times (Table 5). However, there was not a statistical significance between all the molting interval in the two groups.

**Table 5:** Effects of different diets on molting of juvenile mitten crab (means ± SD)

	Groups	
	FO	LO
First molting interval (d)	32 ± 4.72	26.67 ± 6.26
Second molting interval (d)	36 ± 4.85	36.89 ± 5.01
Third molting interval (d)	36.22 ± 4.21	33.89 ± 3.98

### Digestive enzyme activity of juvenile mitten crab fed either FO or LO diets

The activities of four digestive enzymes were measured to investigate the effects of different lipid diets on the digestion of the crabs. From the results in Table 6, the trypsin activity of crabs fed with LO was significantly lower than crabs fed with FO, but the pepsin activity in LO was significantly higher than the crabs fed with FO. Comparing with protease above, the activities of lipase and amylase were at the same level in the crabs fed with FO and LO.

**Table 6:** Effects of different diets on digestive enzyme activity in hepatopancreas of juvenile mitten crab (means ± SD)

	Groups	
	FO	LO
Trypsin activity (U/mg)	752.03 ± 20.55^b^	520.7 ± 8.01^a^
Pepsin activity (U/mg)	0.24 ± 0.03^a^	0.44 ± 0.06^b^
Lipase activity (U/g)	2.33 ± 0.18	2.16 ± 0.15
Amylase activity (U/mg)	0.29 ± 0.04	0.32 ± 0.02

^a, b^Values within a row having different superscript letters means a significant difference at *P* < 0.05

### Fatty acid composition of the experiment diets and crab hepatopancreas

The fatty acid composition of the diets and the hepatopancreas of the crabs are shown in Table 7. A higher proportion of the fatty acids 14:0, 16:0, EPA, and DHA were present in the FO diet. The fatty acids 18:2n-6 and 18:3n-3 formed a higher proportion of the lipids in the LO diet. The 18:2n-6 and 18:3n-3 fatty acids were significantly higher in the hepatopancreas of crabs fed the LO diet compared to the FO diet. Meanwhile, EPA and DHA were significantly higher in crabs fed the FO diet than the LO diet, indicating that the fatty acid composition of the hepatopancreas was closely related to that of the diets.

**Table 7:** Fatty acid composition (% of total fatty acids) of the experiment diets and crab hepatopancreas

Fatty Acids	Experiment diets		Hepatopancreas	
	FO	LO	FO	LO
C_14:0_	4.91	1.63	3.76 ± 0.16^b^	2.19 ± 0.04^a^
C_15:0_	0.91	0.33	0.99 ± 0.63^b^	0.63 ± 0.02^a^
C_16:0_	16.93	11.88	17.11 ± 0.80^b^	15.27 ± 0.45^a^
C_18:0_	5.16	5.68	3.80 ± 0.05^a^	4.37 ± 0.13^b^
C_20:0_	0.65	0.21	0.28 ± 0.03	0.37 ± 0.17
SFA	28.95	20.39	25.94 ± 0.93^b^	22.84 ± 0.42^a^
C_14:1n-5_	0.18	0.12	0.82 ± 0.03^b^	0.41 ± 0.02^a^
C_16:1_	4.74	0.47	9.62 ± 0.40^b^	6.93 ± 0.03^a^
C_18:1n-9_	13.46	17.36	24.21 ± 0.73^a^	25.95 ± 0.60^b^
C_18:1n-7_	2.63	1.30	3.67 ± 0.01^b^	3.16 ± 0.13^a^
C_20:1n-9_	2.65	0.31	1.88 ± 0.10^b^	0.74 ± 0.07^a^
C_22:1n-9_	3.62	0.22	1.26 ± 0.28	–
MUFA	27.29	19.80	41.46 ± 0.73^b^	37.19 ± 0.39^a^
C_18:2n-6_	11.97	17.96	10.19 ± 0.12^a^	15.13 ± 0.24^b^
C_18:3n-3_	2.63	32.29	1.76 ± 0.04^a^	15.95 ± 0.28^b^
C_20:2n-6_	0.19	0.12	0.67 ± 0.03^a^	0.77 ± 0.03^b^
C_20:4n-6_	0.72	0.12	0.73 ± 0.05	0.60 ± 0.40
C_20:5n-3_	7.10	0.00	3.97 ± 0.16^b^	0.75 ± 0.04^a^
C_22:6n-3_	10.50	0.13	6.02 ± 0.53^b^	1.00 ± 0.06^a^
PUFA	34.30	52.48	23.35 ± 0.70^a^	34.19 ± 0.09^b^
HUFA	19.18	1.08	11.40 ± 0.71^b^	3.12 ± 0.50^a^
n-3PUFA	20.98	32.83	11.76 ± 0.66^a^	17.69 ± 0.20^b^
n-6PUFA	13.32	19.65	11.59 ± 0.08^a^	16.49 ± 0.20^b^

^a, b^Values within a row having different superscript letters means a significant difference at *P* < 0.05

### Identification of the proteins obtained from proteomic analysis

**Fig. 1:**
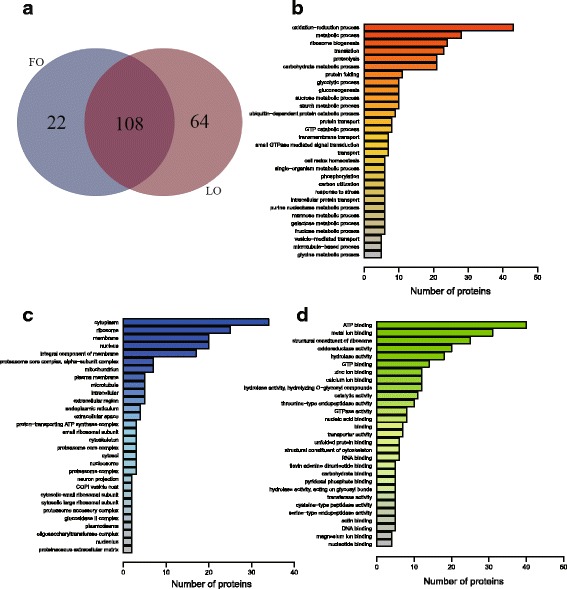
Differentially expressed proteins and gene ontology classification. **a** The differentially expressed proteins in crabs fed with FO and LO and the same differentially expressed proteins in FO and LO. **b, c** and **d** GO annotation of the all the proteins identified from the proteomic analysis of the crab hepatopancreas fed with FO and LO. **b** biological process, **c** cellular component, **d** molecular function

A total of 716 proteins were obtained from the proteomic analysis. GO annotation of the proteins indicated that the proteins were mainly enriched in oxidation-reduction process, metabolic process, and ribosome biogenesis for biological process (Fig. 1b). As for cellular component, cytoplasm, ribosome, membrane, and nucleus were enriched significantly (Fig. 1c). While in molecular function were the ATP binding, metal ion binding, and structural constituent of ribosome (Fig. 1d).

### Identification of differential abundant hepatopancreas proteins

Analysis of protein quantification identified 194 proteins as differentially expressed in FO and LO (Additional file 1: Table S1). Compared to crabs fed the FO diet, 43 proteins were significantly upregulated, and 65 proteins were downregulated in crabs fed with LO diet. A total of 64 proteins were only identified in crabs fed with LO while 22 proteins were only identified in crabs fed FO (Fig. 1a). Heat shock proteins were highly or only expressed in crabs fed the LO diet. In contrast, 4 proteins involved in lipid metabolism including fatty acid synthase (FAS), long-chain fatty acid transport protein 4 (FATP4), acyl-CoA delta-9 desaturase, and fatty acid-binding protein 1 (FABP1), were highly expressed in FO.

### KOG classification of the differentially expressed proteins

**Fig. 2:**
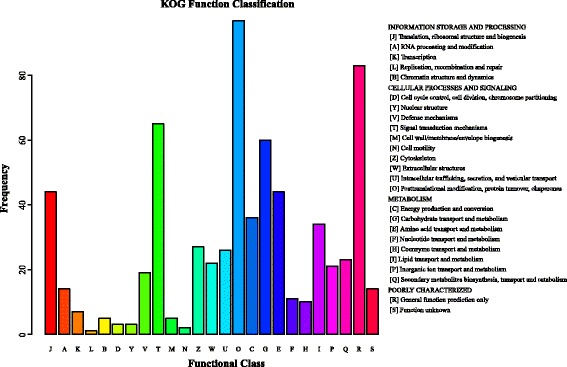
KOG functional classification of differentially expressed hepatopancreas proteins between crabs fed FO and LO diets

The 194 proteins were classified into 25 KOG functional classifications (Fig. 2). Among these classifications, the “posttranslational modification, protein turnover, chaperones” represented the largest group, followed by “general function prediction only”, “signal transduction mechanisms”, “carbohydrate transport and metabolism”, and “translation, ribosomal structure and biogenesis”.

### Analysis of gene expression by real-time PCR and PRM

**Fig. 3:**
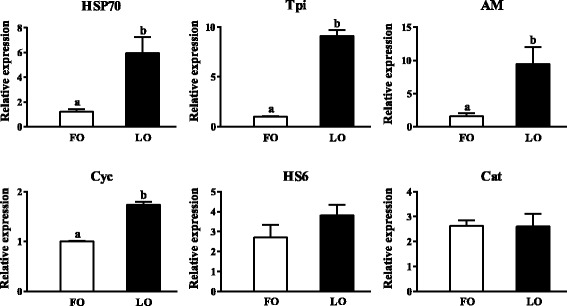
The gene expression in the hepatopancreas of six genes analyzed by RT-PCR between crabs fed FO and LO diets. HSP70: heat shock protein 70; Tpi: triosephosphate isomerase; AM: alpha 2-macroglobulin; Cyc: cyclophilin A; HS6: hemocyanin subunit 6; Cat: cathepsin C

Six proteins significantly increased in crabs fed the LO diet were randomly selected to validate the proteomic data at the mRNA level. The gene expression of all 6 proteins were upregulated in crabs fed the LO diet, and was statistically significant in 4 proteins (Fig. 3). From Table 3, we could find that the FC of the differently expressed protein in PRM was in agreement with proteomic analysis.

## Discussion

The sharply-increasing price of fish oil has prompted research into the use of vegetable oil in replacing fish oil in the diet of crustaceans. In *Litopenaeus vannamei*, the replacement of fish oil by 75% soybean oil did not affect the weight gain rate and SGR of the shrimp [32]. The complete replacement of fish oil might even increase weight gain, SGR, and survival in *Cherax quadricarinatus* and juvenile *Macrobrachium nipponense* [33, 34]. However, in juvenile *L. vannamei*, contrary results were obtained [35]. In the present study, the replacement of fish oil with linseed oil in the diet of mitten crab did not affect survival, weight gain, carapace length, carapace width, SGR, or hepatosomatic index. The same results have been reported by Chen [36]. From the results above, we could find that no effects were found as the replacement of the FO by vegetable oils in most freshwater species. Comparing with marine species, most freshwater species have lower HUFA demand [36]. Mitten crab spend most of its life in freshwater, and as an omnivorous species, thus making it easier to adapt the replacement of FO by vegetable oils in diets [37].

Comparing the two diets in the present study, the fish oil had a higher content of n-3 HUFAs, including EPA and DHA, while the linseed oil contained a high proportion of linolenic acid. It had been reported that n-3 HUFAs have an important role in the carapace development of crabs. In the larval and juvenile *Scylla serrata*, diet lacking in HUFAs led to reduced carapace width due to the lack of the ability to bioconvert C18 unsaturated fatty acids to HUFA [3, 38]. In contrast, in the present study, carapace length and carapace width were not reduced in crabs fed the LO diet lacking in HUFAs. A potential explanation for this is a difference in the ability of *S. serrata* and *E. sinensis* to bioconvert C18 unsaturated fatty acids to HUFAs; mitten crab may have the ability to convert linoleic acid and linolenic acid to EPA and DHA [39]. This ability had been confirmed in other crustaceans such as *Penaeus chinensis* and *Penaeus japonicus* [40, 41].

The utilization of the principal nutrients was largely determined by the activity of digestive enzymes [42, 43]. And many studies had demonstrated that the activity of digestive enzymes in crustaceans were regulated by many factors, one of which was the dietary nutrients [42, 44–46]. There were studies indicated that the activity of digestive proteases could be changed by the dietary lipid and protein level [42, 43, 47]. The results indicated that the activity of proteases could be significantly changed by the dietary lipid sources, high content dietary HUFAs could promote the activity of trypsin, but inhibit the activity of pepsin. Dietary fatty acids didn’t have any effects on the activities of lipase and amylase. Lipid was the main dietary sources for mitten crab to obtain energy for the growth and development. A lot of energy was required for the molt in the life history of mitten crab, thus a relatively stabilized activity of lipase was needed for the crustaceans to obtain enough energy at the same dietary lipid level [48].

The high content of EPA and DHA in the mitten crab was the main value of this species, great efforts have been made to find the HUFA biosynthesis ability of mitten crab. At present, we have obtained 4 kinds of enzyme which participated in the HUFA biosynthesis, including two kinds of desaturase and two elongases [49–52]. These enzymes were relative to the desaturation of C18 fatty acid, and the elongation of C16 saturation and monounsaturated fatty acid [53, 54]. Never had an enzyme was found in mitten crab relative to the biosynthesis of HUFA, such as EPA and DHA. In this study, EPA was not found in the diet of LO, but 0.75% EPA was found in the hepatopancreas of the crabs fed with LO. Thus we speculate that mitten crab maybe have the biosynthesis ability of EPA using other fatty acids.

From the proteome, we identified 3 heat shock proteins (HSPs) which were highly expressed only in crabs fed the LO diet. HSPs are key proteins in regulating the heat shock response to cope with both internal and external environmental stress [55, 56]. In crustaceans, studies indicate that the expression of HSPs are significantly upregulated after a bacterial challenge or when experiencing stress [57–59]. The increased expression of the HSPs HSP10 and HSP60 may indicate greater inflammation or bacterial infection in crabs fed the LO diet.

Four proteins involved in lipid metabolism were highly expressed in crabs fed the FO diet. FAS is a multi-enzyme protein that synthesizes saturated fatty acids, such as palmitic acid (16:0) and stearic acid (18:0), from acetyl-CoA [60]. It had been previously reported that both EPA and DHA suppress the expression of FAS at the level of transcription [61, 62]. In contrast, in the present study the abundance of FAS was significantly higher in FO than LO. This may be due to the presence of other fatty acids in the FO besides EPA and DHA, including tetradecanoic acid (14:0), which was present in high amounts in the FO, and could be transformed to the substrate of FAS.

Acyl-CoA delta-9 desaturase introduces a double bond in 16:0 and 18:0 and is a rate-limiting enzyme in the biosynthesis of monounsaturated fatty acids [63]. Acyl-CoA delta-9 desaturase was isolated from mitten crab in 2013 [50]. In agreement with our results, it has been reported that the substitution of fish oil with soybean oil decreases acyl-CoA delta-9 desaturase mRNA expression [63]. According to the fatty acid composition of FO and LO, we could find that C16:0, the substrate of acyl-CoA delta-9 desaturase, was significantly higher in FO than LO. Therefore, we speculated the higher abundance of acyl-CoA delta-9 desaturase in FO was due to the high content of 16:0 in FO.

Two proteins involved in transport of fatty acids significantly increased in crabs fed FO compared to the LO diet: FABP1 and FATP-4 [64, 65]. It had been reported that different FATP proteins preferentially transport different fatty acids [66] and correlations have been reported between FATP-4 mRNA expression and n-3 long-chain polyunsaturated fatty acids in human placenta [67]. In mitten crab, Es-FABP has been shown to have a role in lipid transport during rapid ovarian growth [68]. In the present study, FATP-4 was only found in FO, and FABP1 abundance was higher in FO compared to the LO diet. This suggests that the presence of HUFAs in the diets of mitten crab could increase the protein abundance relative to the transport of the HUFAs, thus laying the foundation of the high content of HUFAs in the hepatopancreas of mitten crab.

## Conclusions

From the results in the present study, we conclude that the replacement of FO with LO did not have significant effects on the growth performance and molting of juvenile mitten crab. Trypsin activity of crabs fed with LO was significantly lower than crabs fed with FO, but the pepsin activity in LO was significantly higher than the crabs fed with FO. The activity of lipase and amylase didn’t have significant change. The fatty acid composition of the hepatopancreas reflected the fatty acid composition of the diets. From the proteome, we could conclude that the replacement of FO with LO may decrease the ability of mitten crab to cope with stress. The effects of the replacement on the lipid metabolism were focused on the synthesis and transport of fatty acids. The presence of HUFAs in the diets of mitten crab could increase the protein abundance relative to the transport of the HUFAs, thus laying the foundation of the high content of HUFAs in the hepatopancreas of mitten crab.

## Additional file

Additional file 1: **Table S1.** Differentially expressed proteins in the hepatopancreas of crabs fed with FO and LO. (XLSX 58 kb)

## Abbreviations

18S: 18S ribosomal RNA
AM: Alpha 2-macroglobulin
Cat: Cathepsin C
Cyc: Cyclophilin A
DHA: Docosahexaenoic acid
DTT: DL-Dithiothreitol
EPA: Eicosapentaenoic acid
FABP1: Fatty acid-binding protein 1
FAS: Fatty acid synthase
FATP4: Long-chain fatty acid transport protein 4
FCR: Feed conversion ratio
FDR: False discovery rate
FO: Fish oil
GO: Gene ontology
HPLC: High performance liquid chromatography
HS6: Hemocyanin subunit 6
HSP: Heat shock protein
HSP70: Heat shock protein 70
HUFA: Highly unsaturated fatty acid
KOG: EuKaryotic orthologous groups
LO: Linseed oil
S: Surviving rate
SDS-PAGE: Sodium dodecyl sulfate polyacrylamide gel electrophoresis
SGR: Specific growth rate
Tpi: Triosephosphate isomerase
WG: Weight gain
